# Clearing the air: evaluating institutions’ social media health messaging on wildfire and smoke risks in the US Pacific Northwest

**DOI:** 10.1186/s12889-024-17907-1

**Published:** 2024-02-05

**Authors:** Catherine E. Slavik, Daniel A. Chapman, Alex Segrè Cohen, Nahla Bendefaa, Ellen Peters

**Affiliations:** 1 School of Journalism and Communication, University of Oregon, 1715 Franklin Boulevard, Eugene, OR 97403 USA; 2 Center for Science Communication Research, University of Oregon, Eugene, OR USA; 3 Department of Psychology, University of Oregon, Eugene, OR USA

**Keywords:** Wildfire smoke, Public health, Risk communication, Social media, Protection motivation theory, Environmental health

## Abstract

**Background:**

Wildfire smoke contributes substantially to the global disease burden and is a major cause of air pollution in the US states of Oregon and Washington. Climate change is expected to bring more wildfires to this region. Social media is a popular platform for health promotion and a need exists for effective communication about smoke risks and mitigation measures to educate citizens and safeguard public health.

**Methods:**

Using a sample of 1,287 Tweets from 2022, we aimed to analyze temporal Tweeting patterns in relation to potential smoke exposure and evaluate and compare institutions’ use of social media communication best practices which include (i) encouraging adoption of smoke-protective actions; (ii) leveraging numeric, verbal, and Air Quality Index risk information; and (iii) promoting community-building. Tweets were characterized using keyword searches and the Linguistic Inquiry and Word Count (LIWC) software. Descriptive and inferential statistics were carried out.

**Results:**

44% of Tweets in our sample were authored between January-August 2022, prior to peak wildfire smoke levels, whereas 54% of Tweets were authored during the two-month peak in smoke (September-October). Institutional accounts used Twitter (or X) to encourage the adoption of smoke-related protective actions (82% of Tweets), more than they used it to disseminate wildfire smoke risk information (25%) or promote community-building (47%). Only 10% of Tweets discussed populations vulnerable to wildfire smoke health effects, and 14% mentioned smoke mitigation measures. Tweets from Washington-based accounts used significantly more verbal and numeric risk information to discuss wildfire smoke than Oregon-based accounts (*p* = 0.042 and *p* = 0.003, respectively); however, Tweets from Oregon-based accounts on average contained a higher percentage of words associated with community-building language (*p* < 0.001).

**Conclusions:**

This research provides practical recommendations for public health practitioners and researchers communicating wildfire smoke risks on social media. As exposures to wildfire smoke rise due to climate change, reducing the environmental disease burden requires health officials to leverage popular communication platforms, distribute necessary health-related messaging rapidly, and get the message right. Timely, evidence-based, and theory-driven messaging is critical for educating and empowering individuals to make informed decisions about protecting themselves from harmful exposures. Thus, proactive and sustained communications about wildfire smoke should be prioritized even during wildfire “off-seasons.”

**Supplementary Information:**

The online version contains supplementary material available at 10.1186/s12889-024-17907-1.

## Background

Wildfire smoke contributes substantially to the global disease burden, and it is getting worse with climate change [1, 2]. The Pacific Northwest region of the United States (US) has seen an especially large increase in the quantity and scope of wildfires, and populations there already face increased risks of health harms attributable to smoke exposure [3]. The region’s poor air quality due to wildfire smoke is a significant and growing public health challenge [4].

Wildfire smoke contains several compounds hazardous to health, such as Fine Particulate Matter (PM_2.5_), carbon monoxide, nitrogen oxides, methane, trace metals, and carcinogens like formaldehyde, polycycle aromatic hydrocarbons, and acrolein [5]. Although adverse health impacts associated with wildfire smoke can affect all populations, sensitive groups like children, pregnant people, and those with existing respiratory and cardiovascular conditions are especially at risk [6].

To prevent diseases attributable to wildfire smoke, public education and risk communication about smoke risks and exposure mitigation measures are needed [7]. Smoke education and communication efforts require a variety of actors, each responsible for communicating specific types of information to particular audiences [8]. Smoke travels across geographic boundaries following wind patterns, and thus, can impact air quality in places far from the original source [9]. As a result, communications emerge from local, regional, and national sources. In addition, since air quality risks are both environmental *and* public health issues, government agencies and communicators from both domains tend to undertake risk communication activities around wildfire smoke [10].

During wildfire events, individuals have displayed a keen sense of place and seek region-specific updates concerning fire impact and wildfire smoke [11, 12]. While conveying health risks across diverse geographic areas presents obstacles, populations gain from messages that highlight the unique conditions of an ongoing emergency, and from information that feels genuine and are useful to them [13]. Some evidence also suggests that the public trusts local sources of wildfire smoke information more than state-level or federal-level sources [14]. Despite large demand for hyper-local wildfire risk information in the US, effective communications that promote risk comprehension and awareness of exposure mitigation measures are lacking [15].

Twitter (now “X”) is a popular social media platform used by government agencies and officials to disseminate information, providing citizens with a direct link to those leading the response to environmental hazards and public health emergencies [16–18]. In the US, most federal government officials have had a Twitter account, and some sources indicate that about a quarter of the public used Twitter as recently as 2021 [19, 20]. Twitter is a common tool for health promotion and risk communication [21, 22], and in some areas of the US Pacific Northwest—a region that includes the states of Washington and Oregon—the platform has served as a popular means for citizens to express wildfire smoke concerns, seek updates on risks, and learn about intervention strategies [23, 24]. In these states, the percentage of adults with a Twitter account was estimated to be 27% in 2021 [25]. Van Deventer et al. found that the majority of government-authored communications about wildfire smoke in this region were disseminated via Twitter and other social media platforms [10]. An active social media presence is increasingly viewed as important for officials to communicate smoke risks to communities proactively [26]; however, in practice, advice from governments on smoke-protective actions is often reactive and/or comes too late after a smoke event [10, 27, 28]. The US Environmental Protection Agency (EPA) recommends that officials communicate smoke risks and instruct households on what preparations to make during wildfire off-seasons [29].

Prior research studying institutional health messaging on Twitter and communication best practices found that Tweets authored by public health organizations generally served at least one of three message functions [30, 31]. These message functions, originally conceptualized by Lovejoy and Saxton, can be classified based on whether Tweets: (i) encourage members of the public to adopt an action or behavior (“action”); (ii) provide information to the public (“information”); or (iii) promote community-building, give recognition and thanks to community members, or otherwise signal community engagement (“community”) [32].

Although limited studies have tied social media communications like Tweets directly to healthy behavior change [33, 34], governments increasingly consider their communications to be key for motivating the public to take individual actions that reduce exposure to wildfire smoke [35]. One way to encourage action-taking may be through application of constructs from Protection Motivation Theory, which is a model of disease prevention in social psychology and health promotion. The theory posits that people’s intentions to protect themselves from harm are influenced by four cognitions: risk severity, likelihood of experiencing harm, effectiveness of mitigative measures to protect from harm, and the belief, known as self-efficacy, that one can successfully execute these measures [36]. Health agencies and officials have employed messaging based on these Protection Motivation Theory cognitions in public education campaigns [37–39], and their use has been shown to increase people’s intentions to take action to avoid harm from a variety of environmental health hazards [40–42]. Although the application of Protection Motivation Theory constructs in public health messaging on Twitter has not been studied directly, previous studies analyzing communications authored by public health agencies have found that “action” Tweets, which may bolster self-efficacy beliefs, and Tweets specifically referencing hazard severity, garner higher engagement from users [17, 43].

Institutional health communications that primarily serve to inform users about a hazard also have an important function on Twitter [44], particularly during wildfire events [45]. Best practices in health risk communication point to using numeric information to promote accurate perceptions of risks, as people seem to prefer receiving risk information that contains numbers (especially the highly numerate) [46–48], or that contain numbers in combination with verbal labels [49, 50]. They also find messages with numbers more useful. In one study, participants found websites with numeric information clearer and more useful than a site without it, and they were also more motivated to use the information [51]. This preference for numbers may be partly attributed to the imprecision of risk information when expressed verbally, for example, words like “significant” may lead to varying interpretations among individuals [52].

Additionally, numerical risk information may increase intentions to act to reduce environmental risks [53], as it increases other health-protective intentions [54, 55]. Health risks attributable to poor air quality are generally communicated by referencing a specific hazard category from the US Environmental Protection Agency’s Air Quality Index (AQI), which helps the public evaluate how hazardous the air is on a scale from 0 (good) to 500+ (hazardous) [56]. Importantly, using interpretative labels to denote different categories of risk on a scale has been found to aid people’s health decision making and their interpretation of numeric information [57]. Some research also suggests health-based AQI risk labels like those used in the US (e.g., unhealthy, hazardous, etc.) are more effective at motivating protective action intentions than non-health-based air quality risk labels (e.g., poor, polluted, etc.) [58]. However, to our knowledge, only one previous study has explored how institutions or officials inform the public about air quality health risks on Twitter during wildfire smoke events [10].

Institutional use of social media like Twitter centers around engaging users and building (virtual) communities. It is generally viewed as an important way to promote public participation in health promotion and decision making [59]. Some evidence suggests that encountering Tweets about community-building or social practices like care and compassion can influence the adoption of prosocial behaviors [60]. Further, communications encouraging dialogue between community members and public engagement may increase trust and other favorable perceptions of institutions and government organizations [61], which have been found to increase people’s intentions to adhere to public health recommendations during events like natural disasters [62]. Despite this, health agencies appear to author fewer Tweets that focus on “community”, relative to the “action” and “information” functions [30, 31].

The literature reviewed above suggests that extensive research points towards how to craft effective social media messages about a variety of health risks. However, our understanding of the actual communication practices of governments—especially as they pertain to wildfire smoke—remains limited. This study aimed to: [1] analyze temporal Tweeting patterns in relation to potential wildfire smoke exposure in Washington and Oregon, and; [2] evaluate and compare institutions’ use of three best practices for communicating on social media. Based on our findings, we generated practical recommendations for public health practitioners and researchers communicating about wildfire smoke risks on social media. Each Tweet in our dataset was coded for language that could encourage action-taking, its use of risk information, and for language that could promote community-building. The research questions we sought to answer were:

1. Do temporal Tweeting patterns about wildfire and smoke align with daily average AQI values?
2. Do institutional Tweets about wildfires and smoke apply social media communication best practices, which:
  - Encourage the adoption of protective actions for reducing smoke exposure based on predictions from Protection Motivation Theory?
  - Inform users about the health risks associated with smoke exposure by leveraging verbal cues, numeric information, and AQI risk labels?
  - Promote community-building through references to social interactions and/or social behaviors?

3. How does the use of health messaging across these dimensions compare by institutional characteristics including type, regional scale, and location?

## Methods

### Tweet retrieval

An initial scoping review of online information sources identified key government institutions and agencies serving as official disseminators of air quality information to citizens residing in Oregon and Washington. Relevant institutions included national and state-level environmental and health agencies, as well as local health departments. Using the Twitter interface to manually search the names of these government organizations, 34 Twitter accounts were identified from which to draw the sample of Tweets from (See Supplemental Table 1). This study did not include Tweets from organizations related to wildfire emergency response, such as local fire departments, as these accounts tended to disseminate information about fire spread and evacuation notices as opposed to health-related information about wildfires and air quality. Of note, Twitter was renamed “X” in July 2023 several months after data collection for this study had taken place.

Twitter data was downloaded in January 2023 from the 34 Twitter accounts selected for this study using a Twitter Application Programming Interface (API) accessed through R using the ‘rtweet’ package [63]. An R script was developed to download the maximum number of Tweets from each account (i.e., the most recent 3,200 Tweets) permitted for account-specific searches required by Twitter’s API. The download yielded a dataset of 85,406 Tweets authored by the 34 accounts of interest published between April 2009 and January 2023. We limited our analysis to include only Tweets authored between January 1st, 2022, and December 31st, 2022, as this represented the most complete dataset; all but two Twitter accounts (out of 34) ‘Tweeted’ during this time, resulting in 24,430 Tweets authored by 32 accounts.

We further restricted our analysis to Tweets about wildfires and smoke, based on whether they contained the keywords “smoke” or “fire”, and excluded any Tweets containing the words “tobacco”, “cigarette” or “secondhand”. Tweets that did not contain any text (e.g., contained an image only), would have also been screened out at this stage. This step resulted in a sample of 1,879 Tweets. Next, two coders worked together to screen out any Tweets not about wildfires or smoke that remained in the dataset. The two coders engaged in discussions, deliberating on any coding discrepancies between individual Tweets until they reached a consensus. Tweets screened out at this stage included, for example, those discussing air pollution from household fireplaces or wood stoves, or air pollution due to fireworks (*N* = 593). Wood-heating in homes is common across many regions of the US Pacific Northwest during the winter months and can account for the majority of smoke produced (and the PM_2.5_ recorded) during that season [64]. Tweets that referenced air pollution due to forest management fires (e.g., prescribed forest burns) were retained in our analysis, yielding a final dataset—authored by 30 different accounts—of 1,287 Tweets about wildfires and smoke.

### Twitter account classification

The 30 Twitter accounts that authored the Tweets in our dataset were classified based on three categorical variables. First, an account’s location was either classified as Washington (WA) (*N* = 20 accounts, *N* = 830 Tweets), Oregon (OR) (*N* = 7 accounts, *N* = 323 Tweets) or USA-based (*N =* 3 accounts, *N* = 134 Tweets) based on whether the institution was located in one of the two states or belonged to an account attached to a national agency (e.g., the US EPA’s Office of Air and Radiation), respectively. Second, an account was classified by its regional scale as either local (*N* = 21 accounts, *N* = 384 Tweets) or as a state-level or national-level account (*N* = 9 accounts, *N* = 903 Tweets) based on whether the account served citizens from a county, or citizens across an entire state (or multiple states), respectively. For example, Twitter accounts classified as local would have included local public health departments (e.g., Seattle and King County’s @KCPubHealth account), while Twitter accounts classified as regional would have included both state agencies (e.g., Oregon’s Department of Environmental Quality @OregonDEQ) and national agencies (e.g., US EPA’s AirNow Program @AIRNow). Lastly, accounts were classified as either environmental (*N* = 10 accounts, *N* = 882 Tweets) or health (*N* = 20 accounts, *N* = 405 Tweets) based on their institutional mandate.

### AQI data retrieval and analysis

This study used daily AQI values for PM_2.5_ to indicate potential wildfire smoke exposure. Exposure to PM_2.5_ from wildfire smoke has been associated with increased incidences of all-cause mortality and respiratory morbidity, including exacerbations to asthma and COPD, pneumonia and bronchitis [65]. Further, Burke et al. previously found that wildfire smoke caused more than 75% of exceedances in daily PM_2.5_ concentrations in Washington and Oregon between 2020 and 2022 [66]. Thus, PM_2.5_ is frequently used as an indicator of wildfire smoke exposure. Daily air quality summary statistics for PM_2.5_ in Oregon and Washington states were downloaded from the US EPA for the year 2022 to examine temporal trends in both states’ levels of wildfire smoke [67]. The data is based on the EPA’s Air Quality System, which leverages air quality measurements for various criteria pollutants from multiple state monitoring sites and undergoes validation. Daily maximum AQI values (ranging from 0 to 500 based on the US AQI) were aggregated by state and by month.

### Protection motivation theory scoring and analysis of tweets

We searched for keywords associated with each of the four dimensions of Protection Motivation Theory [36] to devise a total score out of 7 for each Tweet (see Table 1). The first dimension, “severity”, was assessed using three sub-dimensions. For the first sub-dimension, “consequences”, a Tweet would receive a score of 1 if it mentioned any health consequence or health effect associated with wildfire smoke exposure (e.g., asthma, coughing, etc.). For the second sub-dimension, “threat”, a Tweet would receive a score of 1 if it mentioned words associated with wildfire smoke threats to health (e.g., PM_2.5_, pollution, etc.). For the third sub-dimension, “magnitude”, a Tweet would receive a score of 1 if it mentioned how severe the threat to health was (e.g., severe, serious, etc.). Thus, a Tweet could receive a maximum score of 3 for the “severity” Protection Motivation Theory dimension if it used messaging satisfying all three of the sub-dimensions.

The second Protection Motivation Theory dimension, “likelihood”, was assessed using two sub-dimensions. For the first sub-dimension, “probability”, a Tweet would receive a score of 1 if it mentioned how likely the threat to health was (e.g., uncertain, predicted, etc.). For the second sub-dimension, “vulnerability”, a Tweet would receive a score of 1 if it mentioned any vulnerable groups more likely to experience health consequences from smoke exposure (e.g., children, pregnant people, etc.). Thus, a Tweet could receive a maximum score of 2 for the “likelihood” Protection Motivation Theory dimension if it used messaging satisfying both of sub-dimensions.

For the third Protection Motivation Theory dimension, “mitigation”, a Tweet would receive a score of 1 if it mentioned any mitigation measures from smoke exposure (e.g., air purifier, staying indoors, etc.). Lastly, for the fourth Protection Motivation Theory dimension, “self-efficacy”, a keyword search for the terms “protect”, “safe” and “can” was carried out; Tweets containing one or more of these words were read to examine the context surrounding the use of the words and whether they were used to describe people’s ability to protect themselves, stay safe or execute some specific action (e.g., “*Learn how you can protect your health from wildfire smoke…*”). The “self-efficacy” dimension of the Protection Motivation Theory score was also scored as a binary variable out of 1, denoting a presence or absence of self-efficacy language.

Protection Motivation Theory scores were created for each Tweet by tallying up the presence of each Protection Motivation Theory dimension/sub-dimension. We followed a similar approach to one adopted by Zhang et al., who also linked keywords from Tweets to specific constructs of a health behavior theory in order to examine the usage of these constructs in health-related Twitter discussions [68]. In this study, each Tweet obtained a Protection Motivation Theory score ranging from 0 (no Protection Motivation Theory dimensions present) to 7 (all Protection Motivation Theory dimensions and sub-dimensions present).

### Risk information scoring and analysis of tweets

In addition to Protection Motivation Theory scores, Tweets were also coded based on the presence or absence of different types of risk information and specifically whether: (i) verbal cues were used; (ii) numeric information was used; and/or (iii) AQI risk labels were referenced. Table 1 highlights which words were used to create a verbal risk information score, where the presence of any single word would lead to a score of 1 for that category. A Tweet received a score of 1 in the numeric category if it contained a number (i.e., any Arabic integer) in reference to a risk quantity relevant to wildfires and/or smoke, for example, describing a percent likelihood of fire spread or the number of acres burning. Finally, a Tweet received a score of 1 for the AQI risk label category if it referenced any one of the six AQI hazard categories commonly used to quantify risks from poor air quality in the US.

**Table 1:** Keyword search strategies for Protection Motivation Theory and risk language scoring by dimension/sub-dimension

Variables for keyword search		Keywords
**Protection Motivation Theory dimensions**	**Sub-dimensions (if present)**	
Severity	Consequences	Asthma; breath*; cardiovascular; chest pain; condition; cough; damag*; disease; effect; eye; headache; health problem; heart; illness; lung; respirat*; throat; wheez*
	Threat	Air quality; AQI; degraded; hazard; partic*; PM; pollut*; risk; threat
	Magnitude	Danger*; extreme; harm*; serious; severe; unhealthy
Likelihood	Probability	Can^ǂ^; chance; could; expect; forecast; frequent; likel*; may; might; possib*; potential; predict; probab*; uncertain; will
	Vulnerability	Asthma; cardiovascular; child; elderly; kid; old; pregnant; respiratory; sensitive; worker
Mitigation	NA	HEPA; indoor; inside; mask; MERV; monitor; outdoor; outside; purifier; respirator; sensors; shelter; space; window
Self-efficacy	NA	Can^ǂ^; protect; safe
**Risk information dimensions**		
Verbal cues	NA	Big; decline; decrease; elevated; fewer; heavy; high; increase; large; less; low; many; more; most; reduce; rise; rising; small; tiny
Numeric information	NA	[number]; %; percent*
Air Quality Index Risk Labels	NA	Good; hazardous; moderate; unhealthy^†^

Note: *Wildcard search queries were carried out on certain words to capture groups of similar words with different endings

^ǂ^Two possible uses of the word ‘can’ existed in this search and were treated as different keywords, first ‘can’ as a verb for possibility (i.e., can be smoky), and second ‘can’ as a verb for ability (i.e., you can take this action to protect yourself)

^†^The word ‘unhealthy’ appears in three of the six Air Quality Index risk labels: Unhealthy for sensitive groups, Unhealthy, Very unhealthy

### Assessment and analysis of tweets about community-building

Further, this study used a dictionary-based approach with Linguistic Inquiry and Word Count software (LIWC-22) to assess linguistic differences in word use about community-building across the Twitter accounts studied. Boyd et al. provides an overview of the development of the LIWC dictionary and the reliability and validity of the various dimensions the software generates [69]. In essence, each LIWC dimension is composed of a select list of dictionary words that have been found to capture its meaning based on an extensive text corpus of approximately 31 million words from various sources (including thousands of Tweets). We selected two dimensions from LIWC-22 relevant to community-building, and ran all Tweets in our sample through the software to give each Tweet two LIWC scores, representing percentages of total words within a text for two dimensions of interest: “social behavior” and “prosocial behavior”. Prior research has found that the social dimensions LIWC uses are a reliable indicator of social connections and closeness [70]. The “social behavior” dimension captures words associated with social interactions and includes terms like “said” and “share”; The “prosocial behavior” dimension captures a subset of words from the “social behavior” category, but specifically targets terms associated with social behaviors that benefit society or promote caring about others, for example, “care” and “thank” [69].

### Statistical analysis

Descriptive statistics were performed for the Protection Motivation Theory scores and the individual dimensions used to describe the presence/absence of these scores across different groupings of interest (i.e., by institution type [environmental vs. health], regional scale [local vs. state-level or national-level], and location [OR vs. WA vs. USA]). Descriptive statistics were also performed to describe the presence/absence of different risk language in the Tweets, as well as differences in the LIWC dimensions, across institution type, regional scale and location.

Models were constructed to test differences between institution types, regional scales, and locations on the outcomes of interest including the Protection Motivation Theory score, risk language, and LIWC score. For the Protection Motivation Theory and LIWC scores, preliminary analyses revealed potential violations of homogeneity of variance and normality of residuals. Therefore, non-parametric statistical tests were employed for the Protection Motivation Theory and LIWC models. To test for differences between institution type and regional scale, Mann-Whitney Wilcoxon tests—the non-parametric equivalent of independent samples t-tests—were used. The results of these models were accompanied by Vargha and Delaney’s ‘A’ as an effect size measure, for which benchmarks recommended by the developers were provided [71].

For differences across the three locations, the Kruskal-Wallis test (i.e., non-parametric one-way analysis of variance) was used, accompanied by post-hoc tests to compare each location using Dunn’s method and the Benjamini-Hochberg *p*-value adjustment for multiple comparisons [72, 73]. Epsilon-squared (*E*^*2*^) was reported as an effect size measure for the Kruskal-Wallis test. As the outcomes were binary for the models of risk language, logistic regression models were employed. Standard logistic regression results with odds ratios as an indicator of effect size were reported. For the model of location, post-hoc pairwise comparison results were reported based on the marginal means, again using the Benjamini-Hochberg *p*-value adjustment for multiple comparisons.

## Results

### Temporal patterns in institutional tweeting and potential smoke exposure

**Fig. 1:**
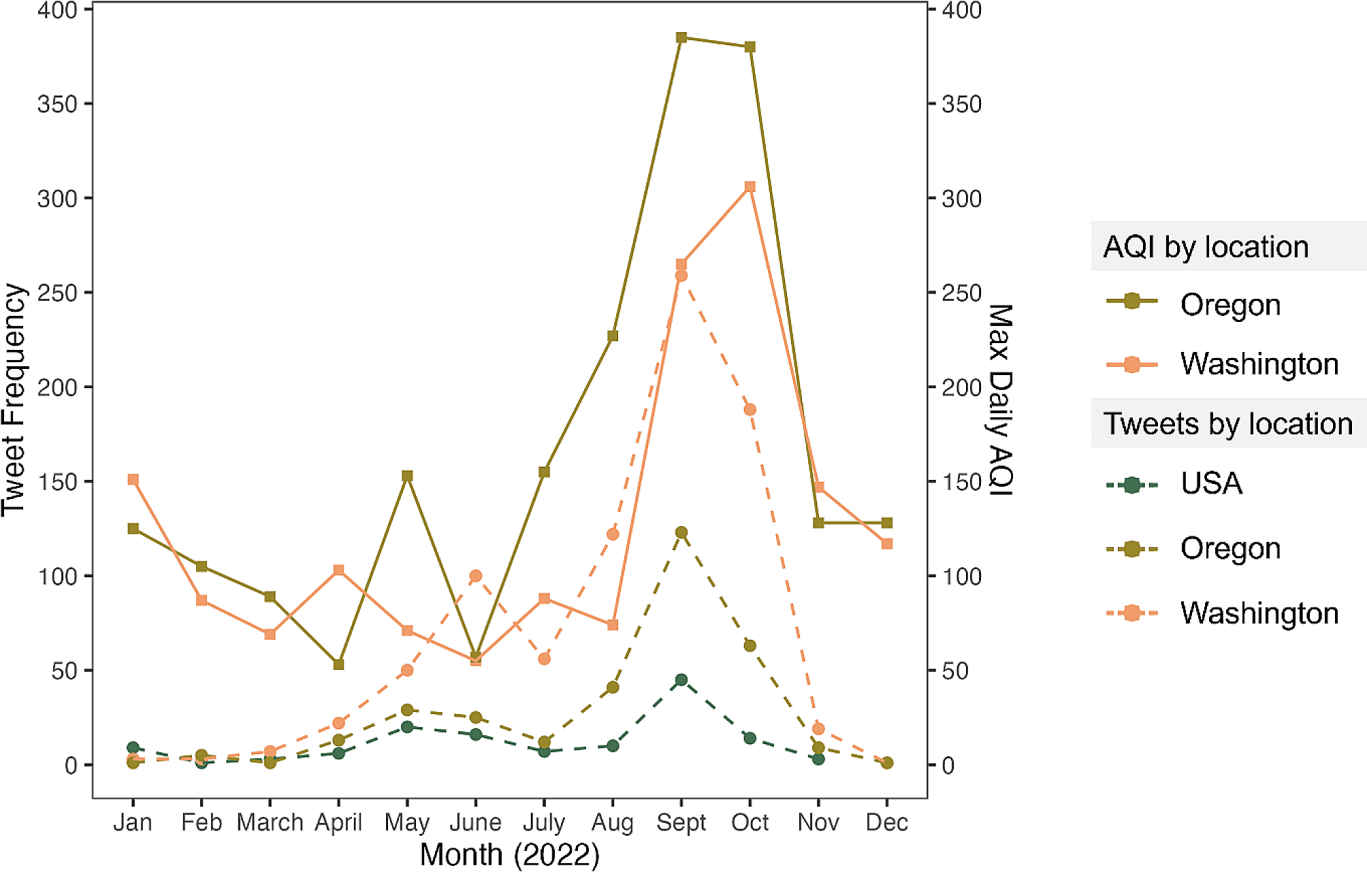
Summed frequencies of wildfire and smoke Tweets authored by institutional accounts (left) and maximum daily AQI values (for PM2.5) (right) in 2022 aggregated by account location and month. *Data sources*: Twitter and US EPA

This study examined whether temporal trends in institutional Tweeting about wildfire smoke corresponded to changes in daily AQI values (i.e., an indicator for potential population exposure to wildfire smoke). Results in Fig. 1 show that values for maximum daily AQI (averaged by month) were highest in September and October 2022 and AQI levels were generally higher in Oregon than in Washington state. Across all accounts, 44% of all Tweets in our sample were authored prior to that year’s wildfire smoke season during the period January to August 2022, while 54% of all Tweets were authored during the two-month peak in smoke levels (during the period September-October 2022) (data not displayed). Both in Washington and also in Oregon, each Twitter account authored on average 18 wildfire-smoke Tweets during the period prior to peak smoke levels, which corresponded to 44% and 39% of each state’s annual total Tweets, respectively. Each USA-based account authored on average 24 wildfire-smoke Tweets during the period of time preceding peak wildfire smoke levels (54% of their annual total Tweets). During the two-month peak period of wildfire smoke, accounts in Washington each authored on average 22 Tweets, while accounts in Oregon appeared to take a more responsive approach and authored an average of 27 Tweets each (54% vs. 58% of their annual total Tweets, respectively). Accounts across the USA each authored an average of 20 Tweets during the peak wildfire smoke period (44% of their annual total Tweets).

### Use of tweets promoting adoption of protective actions from wildfire-smoke exposure

This study explored whether institutional Tweets about wildfire and smoke encouraged the adoption of protective actions to mitigate smoke exposure by applying constructs from Protection Motivation Theory. Results indicate that no Tweet authored by the accounts in the dataset contained all seven Protection Motivation Theory dimensions/sub-dimensions studied (data not displayed). However, most Tweets contained messaging satisfying at least one of the seven Protection Motivation Theory dimensions (*N* = 1054, 82%). Only 15 Tweets used messaging consistent with each of the four Protection Motivation Theory cognitions (i.e., severity, likelihood, mitigation, self-efficacy) by satisfying at least one of three sub-dimensions for “severity”, one of two sub-dimensions for “likelihood”, as well as the “mitigation” and “self-efficacy” dimensions.

Table 2 summarizes the use of Protection Motivation Theory dimensions in the sample of Tweets studied. The most frequently used “severity” sub-dimension in Tweets was “threat” (*N* = 615, 48%), followed by “magnitude” (*N* = 220, 17%). Only 2% of Tweets (*N* = 26) used all three sub-dimensions of the “severity” dimension and 56% of Tweets contained at least one “severity” sub-dimension (*N* = 716) (data not displayed).

**Table 2:** Frequency of use of Protection Motivation Theory health messaging in Tweet sample (*N* = 1,287 Tweets). Percentages add up to more than 100% because a Tweet could be represented in each dimension and sub-dimension

Protection Motivation Theory dimension	Sub-dimension (if present)	Frequency	% of total Tweets
Severity	Consequences	87	7
	Threat	615	48
	Magnitude	220	17
Likelihood	Probability	595	46
	Vulnerability	123	10
Mitigation	NA	178	14
Self-efficacy	NA	305	24

The most frequently used “likelihood” sub-dimension in Tweets was “probability” (*N* = 595, 46%) (Table 2). 10% of Tweets analyzed contained information about vulnerable populations (*N* = 123); The most frequently mentioned terms pertaining to the “vulnerability” sub-dimension were “sensitive” (*N* = 77), “asthma” (*N* = 27), “child” or “kids” (*N* = 30), and “elderly” or “old” (*N* = 25). Less frequently mentioned terms for the “vulnerability” sub-dimension were “pregnant” (*N* = 9), “respiratory” (*N* = 8), “worker” (*N* = 1) and “cardiovascular” (*N* = 1). Only 7% of Tweets (*N* = 88) used both “likelihood” sub-dimensions in their health messaging (data not displayed).

14% of Tweets used messaging to satisfy the “mitigation” Protection Motivation Theory dimension and discussed measures to mitigate exposure to wildfire smoke (*N* = 178) (Table 2). Terms pertaining to staying indoors (“indoor” *N* = 61, “inside” *N* = 14, “shelter” *N =* 13) or limiting outdoor time (“outside” *N* = 34, “outdoor” *N* = 23) were most frequently mentioned. The term “monitor” was also used (*N =* 46). Other mitigation measures referenced in the Tweets included the terms “mask” (*N* = 20), “window” (*N* = 18), “HEPA” or “purifier” (*N* = 12). Nearly a quarter of Tweets used health messaging that satisfied the “self-efficacy” Protection Motivation Theory dimension, which referred to Twitter users’ capacity to execute a smoke-protective behavior (*N* = 305, 24%).

This study also examined how the application of Protection Motivation Theory in Tweets varied by institutional characteristics. No significant differences emerged between institutional use of Protection Motivation Theory dimensions across environmental vs. health institutions (*W* = 177,206, *p* = 0.815, Vargha and Delaney *A* = 0.50) (Table 3). However, a significant difference existed between regional scales; local accounts that serve county-level populations used Protection Motivation Theory health messaging more frequently in their Tweets compared to state- or national-level accounts that serve larger populations (*W* = 200,098, *p* < 0.001, Vargha and Delaney *A* = 0.58).

**Table 3:** Mean Protection Motivation Theory scores by institution type and regional scale

	Protection Motivation Theory score (standard error)	Vargha and Delaney’s A (effect size)	W	*p*
**Institution type**				
Environmental	1.24 (0.04)	0.50 (negligible)	177,206	0.815
Health	1.30 (0.06)			
**Regional scale**				
State or national	1.53 (0.04)	0.58 (small)	200,098	< 0.001
Local	1.92 (0.07)			

Tweets authored by Twitter accounts located in Oregon, Washington, and across the USA significantly differed in their Protection Motivation Theory scores (*X*^2^ (2, *N* = 1287) = 17.17, *p* < 0.001, *E*^2^ = 0.013) (Table 4). Post-hoc comparisons revealed that accounts located in Washington used health messaging with content aligned with Protection Motivation Theory more frequently in Tweets compared to accounts located in Oregon (*p* < 0.001) and accounts across the USA (*p* = 0.053), which did not differ from each other (*p* = 0.552).

**Table 4:** Mean Protection Motivation Theory scores by location with post hoc pairwise comparisons

Protection Motivation Theory score (standard error)			Ε^2^	χ2	*p*	Dunn’s post hoc
**USA**	**OR**	**WA**				
1.51 (0.10)	1.46 (0.07)	1.75 (0.04)	0.013	17.17	< 0.001	USA vs. OR USA vs. WA* WA vs. OR***

Note: *p ≤ 0.05, **p ≤ 0.01, ***p ≤ 0.001

### Use of tweets containing risk information

We further investigated whether institutional Tweets about wildfire and smoke informed people about the health risks associated with smoke exposure through use of verbal risk cues, numeric risk information and AQI risk labels. Three hundred and sixteen Tweets (25% of total sample) included risk information containing some verbal cues in their health messaging (e.g., “*Central WA has experienced fewer smoky days than normal…*”). The term “large” (*N* = 22) was the most frequently used verbal cue for risk information; it quantified the size of fires contributing to wildfire smoke. Just sixty-four Tweets (5%) contained numeric risk language (e.g., “*An AIR QUALITY ALERT is in place for another 48 hours…*”). Two hundred and thirteen Tweets (17%) explicitly used risk language referring to one or more of the AQI risk labels (e.g., “*Air quality has reached the level of unhealthy for sensitive groups across most of #Seattle. Smoke is coming from #BoltCreekFire near Skykomish*”).

This study also explored how accounts’ use of the three types of risk information varied by institutional characteristics. There was no significant difference between environmental and health institutions and their use of verbal risk language (*OR* = 1.13, *p* = 0.360) (Table 5). There was also no significant difference between environmental and health institutions’ use of numeric risk language, (*OR* = 1.07, *p* = 0.812) nor in their use of AQI risk labels, (*OR* = 0.78, *p* = 0.145). Local accounts did not differ in their use of verbal risk language when compared to accounts at the state- or national-level (*OR* = 0.84, *p* = 0.218) (Table 5). However, accounts at a state- or national-level scale were more likely than local accounts to provide numeric risk information (*OR* = 1.89, *p* = 0.05) and less likely to provide AQI risk labels (*OR* = 0.55, *p* < 0.001).

**Table 5:** Counts and likelihood of Tweets containing the three types of risk information studied (verbal cues, numeric information, and AQI risk labels) by institution type and regional scale

	Verbal cues (e.g., “large”)		Odds ratio (95% C.I.)	Numeric information (e.g., “[#]”)		Odds ratio (95% C.I.)	AQI risk labels (e.g., “unhealthy”)		Odds ratio (95% C.I.)
**Institution type**	**Yes**	**None**		**Yes**	**None**		**Yes**	**None**	
Environmental	210	627	1.13 (0.86, 1.48)	43	839	1.07 (0.61, 1.80)	155	727	0.78 (0.56, 1.08)
Health (ref)	106	299		21	384		58	347	
**Regional scale**	**Yes**	**None**		**Yes**	**None**		**Yes**	**None**	
State or national	213	690	0.84 (0.64, 1.11)	52	851	1.89 (1.03, 3.76)	126	777	0.55 (0.41, 0.75)
Local (ref)	103	281		12	372		87	297	

**Fig. 2:**
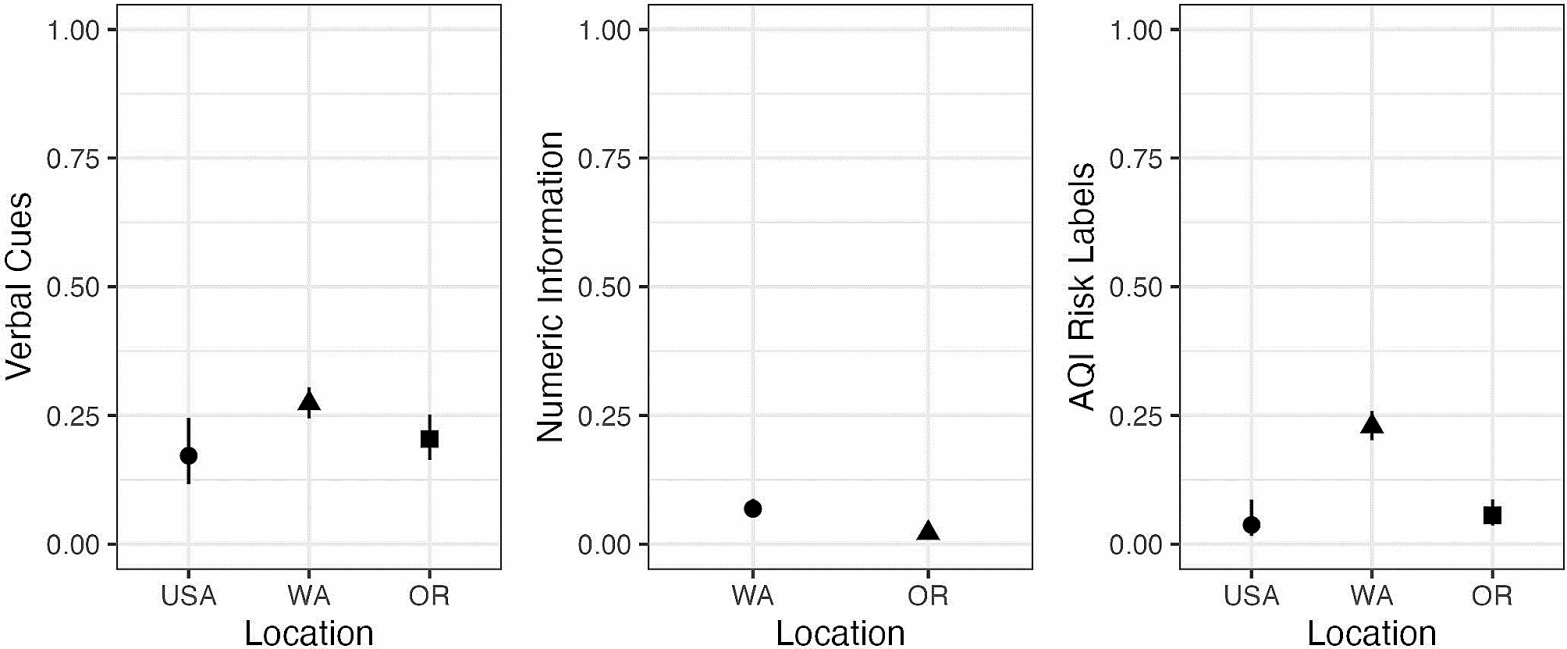
Probability of Tweets containing each of the three types of risk information studied (verbal cues, numeric information, and AQI risk labels) by location. Error bars reflect 95% confidence intervals

There was significant variance predicted by the locations of accounts’ Tweets and their use of verbal risk language (*X*^2^ (2, *N* = 1287) = 10.775, *p* = 0.005). In terms of predicted probabilities, accounts in Washington had a 27.3% probability of using verbal risk language, compared to a 20.4% probability in Oregon accounts and a probability of 17.2% in USA-based accounts (Fig. 2). Pairwise comparisons revealed that Tweets from Washington-based accounts used significantly more verbal risk language than both Oregon (*p* = 0.024) and USA-based accounts (*p* = 0.024), but Tweets from Oregon and USA-based accounts did not differ significantly (*p* = 0.422).

For numeric risk language, none of the USA-based accounts included numeric risk information. Therefore, this model was simplified to compare only Oregon and Washington. There was a significant effect of location such that a greater percentage of Tweets authored by Washington-based accounts included numeric risk language (6.9%) than Tweets authored by Oregon-based accounts (2.2%), (*OR* = 3.33, *p* = 0.003) (Fig. 2).

All three locations (OR, WA, USA) were included in the AQI risk information model. There was significant variance predicted by the different locations for references to AQI risk labels, (*X*^2^ (2, *N* = 1287) = 80.27, *p* < 0.001). Tweets authored by Washington-based accounts had a 22.9% probability of referencing AQI risk labels, accounts in Oregon had a 5.6% probability and the USA accounts had a probability of 3.7% (Fig. 2). Pairwise comparisons revealed that Tweets from Washington-based accounts contained significantly more AQI risk labels than both Oregon-based accounts (*p* < 0.001) and USA accounts (*p* < 0.001); Tweets from Oregon and USA accounts did not differ (*p* = 0.415).

### Use of tweets promoting community-building

This research explored institutions’ use of messaging in Tweets to promote community-building through references to social interactions and social behaviors. Across our sample of Tweets, 1.70% of the words in each Tweet, on average, were words associated with social behaviors (data not displayed). The Tweet with the highest proportion of social behavior words was authored by a local health department located in Washington state, in which 15.38% of the Tweet constituted social process words. Across the whole sample, on average, fewer than 1% of words per Tweet constituted words related to the prosocial behavior dimension (*N* = 0.52%). Out of the 1,287 total Tweets, 611 contained at least one social behavior word (47%), and 236 contained at least one prosocial behavior word (18%).

This study also examined how the use of Tweets promoting community-building varied by institutional characteristics. The LIWC analysis indicated that the use of social behavioral words differed significantly between environmental and health institutions (*p* = 0.024) (Table 6); words associated with this dimension were used more frequently by health institutions compared to environmental institutions. However, the use of words associated specifically with prosocial behaviors (*p* = 0.464) did not significantly differ across institution types. When examining community language use by regional scale, no significant differences were found between local versus state- or national-level institutions’ use of social behavioral (*p* = 0.443) or prosocial (*p* = 0.574) words.

**Table 6:** Mean percentage of words per Tweet referring to community-building by institution type and by regional scale. Means are expressed as the percentage of total words within a Tweet that are words associated with a given LIWC category of community language

LIWC category	Mean word percentage (standard error)		Vargha and Delaney’s A (effect size)	W	*p*
	**Environmental**	**Health**			
Social behavior	1.60 (0.07)	1.92 (0.12)	0.46 (negligible)	165,702	0.024
Prosocial behavior	0.52 (0.04)	0.52 (0.07)	0.51 (negligible)	181,668	0.464
	**State or national**	**Local**			
Social behavior	1.66 (0.07)	1.80 (0.12)	0.51 (negligible)	177,700	0.443
Prosocial behavior	0.53 (0.04)	0.51 (0.07)	0.49 (negligible)	171,059	0.574

As summarized in Table 7, use of words related to social behaviors significantly differed by location (*X*^2^ (2, *N* = 1287) = 91.26, *p* < 0.001) (Table 7). Institutional Twitter accounts based in Oregon, on average, authored Tweets with higher percentages of words associated with social behaviors (2.47%) relative to Tweets authored by accounts located across the USA (2.17%, *p* = 0.043) and accounts based in Washington state (1.33%, *p* < 0.001). Accounts based in Washington authored Tweets containing a significantly smaller proportion of words associated with social behaviors compared to USA-based Twitter accounts (*p* < 0.001). The use of words associated with prosocial behaviors did not differ significantly between account locations (*X*^2^ (2, *N* = 1287) = 0.32, *p* = 0.851) and none of the pairwise comparisons were significant.

**Table 7:** Mean percentage of words per Tweet referring to community language by location. Means are expressed as the percentage of total words within a Tweet that are words associated with a given LIWC category of community language

LIWC category	Mean word percentage (standard error)			Ε^2^	χ2	*p*	Dunn’s Post hoc
	**USA**	**OR**	**WA**				
Social behavior	2.17 (0.22)	2.47 (0.13)	1.33 (0.07)	0.071	91.25	< 0.001	USA vs. OR^*^ USA vs. WA^***^ WA vs. OR^***^
Prosocial behavior	0.57 (0.12)	0.60 (0.08)	0.48 (0.04)	0.0003	0.32	0.851	USA vs. OR USA vs. WA WA vs. OR

Note: *p ≤ 0.05, **p ≤ 0.01, ***p ≤ 0.001

## Discussion

Wildfire smoke is and likely will continue to be a major cause of air pollution in Oregon and Washington [4, 66]. In these regions, environmental and public health agencies are advised to communicate to the public about air quality issues, provide advice on strategies to limit exposure to wildfire smoke, and generally view public education as an important part of their institutional mandate [14, 29, 74]. This research evaluated wildfire and smoke communications authored by Oregon, Washington and USA-based accounts on the social media platform Twitter and examined how health messaging varied by institutions. First, we explored temporal Tweeting patterns in relation to potential smoke exposure. We found that more than half of all Tweets authored by accounts in Oregon and Washington were Tweeted during peak smoke levels (58% and 54% of annual total Tweets, respectively). These results indicated that institutions in Oregon and Washington took a responsive approach to communicate to citizens about wildfire smoke when the risk of exposure was highest. In comparison, the three USA-based accounts Tweeted a smaller proportion of Tweets during peak smoke levels (44% of annual total Tweets). This result may be attributed to the fact that these three accounts represent national agencies that communicate about air quality issues across the USA, and thus, one might expect these accounts to Tweet less frequently in response to specific wildfire and smoke events occurring in a particular region. Still, the findings from this analysis indicate that communicators in Oregon and Washington are generally following the US EPA’s recommendations around communicating about wildfire smoke even during wildfire off-seasons [29]; in both states, each Twitter account authored, on average, the same number of Tweets (18 Tweets) during the period prior to peak smoke levels.

We also examined whether institutions followed best practices for communicating on social media by leveraging three message functions: (i) encouraging the adoption of smoke-protective actions; (ii) informing the public about health risks using verbal cues, numeric information and AQI risk labels; and (iii) promoting community-building. Use of these message functions has been associated with advancing numerous public health goals. For example, messaging encouraging the adoption of protective actions and community-building can lead to changes to behavior that help mitigate health risks associated with exposure to environmental hazards [41, 42, 62], and leveraging numeric, verbal, and AQI risk information appears to aid individuals’ health decision making and understanding of risks [49, 57]. Our findings indicated that institutional accounts used Twitter to promote smoke-related behavior change (as indexed through dimensions of Protection Motivation Theory) more often than they used it to disseminate wildfire smoke risk information or promote community-building.

Specifically, the majority of Tweets we analyzed (82%) included some form of protection motivation constructs—messaging that has been found to generate greater willingness to adopt actions to reduce exposure to hazards [42]. However, less than half of the Tweets in our sample used language associated with community-building (47%), and only a quarter reported any kind of risk information (25% verbal, 5% numeric, 17% AQI). Furthermore, we found differences in institutions’ use of action-, information- and community-based messaging across account types, highlighting the challenges for institutions to communicate about wildfire smoke consistently.

Individuals are increasingly turning to online sources for information about wildfire smoke [23, 24, 27]. Given the limited research evaluating official online wildfire smoke communications [10, 12, 45], our study addressed an important knowledge gap by shedding light on how institutions communicate about wildfire smoke to highly exposed populations through social media. This research also offers lessons for how institutions can improve health messaging about wildfire smoke in the future by leveraging messaging that is evidence-based, timely and theory-driven. As exposures to environmental risks like wildfire smoke become more frequent and more intense with climate change [1], reducing the burden of disease attributable to these hazards will require that health officials not only effectively leverage popular communication channels and distribute relevant communications rapidly, *but also* get the message right. We make four recommendations.

### 1. Institutional messaging requires more integration with protection motivation theory constructs

Individuals and communities can adopt several measures to minimize and mitigate exposure to wildfire smoke, for example, by using respirators and at-home air purifiers, reducing time outdoors during smoke events, and upgrading building ventilation [75]. Since applying constructs from Protection Motivation Theory in health communications has previously been found to encourage action-taking [42], the high frequency of Tweets in our sample containing Protection Motivation Theory messaging indicates that institutions appeared to use language promoting behavior change to reduce the threat of smoke exposure and health harms. This was the case especially among local accounts serving county-level populations, which used this type of messaging more frequently in their Tweets compared to state-level or national-level accounts that serve larger populations. This finding may reflect local health departments being better positioned to implement health promotion interventions and drive behavior change at the community level compared to state and national institutions [31], for example, in organizing clean air spaces during smoke events and encouraging local community members to access them.

Out of the seven Protection Motivation Theory constructs we studied, “threat” and “probability” were most used in Tweets, appearing in nearly half of all Tweets in our sample (48% and 46%, respectively). Thus, institutions appeared to be effective at leveraging certain elements from the Protection Motivation Theory to describe the hazard that smoke poses and the probability of individuals encountering it.

Only 15 Tweets used messaging to satisfy at least some elements from each of the four main Protection Motivation Theory cognitions (i.e., severity, likelihood, mitigation, self-efficacy). Consistent with research by Marfori et al. [27], only 7% of Tweets discussed the health consequences of smoke exposure, which may have impacts on individuals’ abilities to make informed decisions about smoke risks and taking appropriate precautions. Further, the small proportion of total Tweets discussing populations vulnerable to smoke-related health effects and specific mitigation measures (10% and 14%, respectively) represents an important area where health messaging could improve—especially since individuals in vulnerable groups seem to seek-out information about wildfire smoke more frequently and could benefit from tailored messaging [6, 12]. Moreover, increasing people’s perceived vulnerability to wildfire risks, and providing them with actionable solutions to reduce smoke exposure, appear to be important motivators for undertaking risk-mitigating actions [39–41].

Our analysis also found that terms instructing people to stay indoors or limit time outdoors were more frequently mentioned than terms related to other mitigation measures like air purifier use or ventilation systems to improve air quality. This finding was consistent with Van Deventer and colleagues’ research on health messaging in Washington state during a 2018 smoke event [10], suggesting that officials instructed individuals to stay inside without necessarily providing guidance on how to make indoor spaces safer. Since the prevalence of residential heating, ventilation, and air conditioning systems in cities like Seattle, Washington and Portland, Oregon consistently rank lower than many other major US cities [76], and smoke particles can approach 70–100% of the outdoor concentrations in homes without air conditioning [29], staying indoors may not always offer people substantial health benefits and may not be feasible for everyone.

### 2. Combined numeric information, verbal cues, and AQI risk labels should be leveraged in communications about wildfire smoke

The percentage of Tweets in our sample that informed users about *how much* wildfire smoke risk was present using verbal and numeric risk terms (25% and 5%, respectively) was smaller than the percentage of Tweets containing Protection Motivation Theory constructs described above. This finding presents two challenges to risk communicators in Oregon and Washington. First, messaging promoting behavior change appears to be most effective when supplied with information about levels of risk [42], suggesting the frequency of “action” and “information” Tweets should be more similar. Second, since many people appear to prefer to receive risk information containing numbers alone or numbers with verbal cues [49, 50]. The large discrepancy between verbal and numeric risk information reported by institutions in our sample should be addressed by institutions going forward.

Nearly a fifth of Tweets in our sample referenced AQI risk labels to inform audiences about how clean or polluted the air was. As prior research has indicated that risk labels may improve individuals’ health-related judgments and even improve use of numeric information in decisions [57, 77], the use of AQI risk labels in health messaging on wildfire smoke should be continued. Our results indicated that local organizations were more likely to reference AQI risk labels (e.g., “hazardous”) compared to state-level or national-level accounts. This finding can be explained by the fact that AQI information is obtained using local monitors to reflect hazard levels, and thus, Twitter accounts representing individual counties more often report local AQI levels while state-level or national-level accounts likely report broader regional trends in air quality. Since the public generally expects to have access to locally-relevant information about smoke levels in their community [11, 12] and tends to report higher levels of trust in local sources of information [14], local accounts should continue to report local AQI risks in their health messaging when disseminating information about smoke risks. On the other hand, state- or national-level accounts could play a role in re-directing their online audiences to follow local accounts for risk information [31].

### 3. Engaging, bi-directional smoke communications are needed

Our study examined whether institutions encouraged community-building through references to social interactions and/or social behaviors, which may have implications for encouraging public participation in health promotion and trust in government institutions [60, 61]. On average, less than 2% of words in each Tweet in our sample constituted community language. Nearly half the Tweets in our sample (47%) contained at least one word associated with community-building. This proportion of Tweets containing community language is relatively higher than the 22–35% reported in other studies examining public health Twitter communications; however, previous studies relied on manual thematic coding of Tweets, and consequently, direct comparisons are difficult to draw [30, 31]. To assess community language use across various accounts, we used the percentage of words per Tweet containing social behavior words as a more conservative metric summarizing *how much* each Tweet met the community language criteria, rather than only relying on the presence or absence of themes in a text.

Our analysis of community-building messaging by institution did not reveal any significant differences in how various accounts used language about prosocial behavior. However, it did reveal that terms associated with *any* social behavior appeared more in Tweets authored by health institutions compared to environmental institutions. This finding could be explained by the fact that health institutions, focused on community health, appear to leverage more human-centered messaging compared to environmental institutions, which primarily prioritize environmental protection. Since citizens with less knowledge about wildfire risks appear to rely more on community cues as motivation for engaging in wildfire-protective behaviors [41], our finding suggests environmental institutions may benefit from the use of more community language to promote the uptake of AQI messaging. The public’s desire for smoke messaging that engages community members [26] further demonstrates that this is an important message function that officials should prioritize.

### 4. Take advantage of smoke “off-seasons” to implement a proactive approach to wildfire and smoke health messaging

Large-scale wildfires (and wildfire smoke) are not unprecedented in the US Pacific Northwest, and both Oregon and Washington have amassed considerable experience managing public health risks attributable to this hazard [78]. In fact, our analyses indicated that, on average, populations in both states appeared to experience similar levels of smoke exposure in 2022 based on maximum daily AQI values, which was also corroborated by research from Burke et al. [66]. Yet, our study suggests that institutions in both states took slightly different approaches to communicate about wildfire and smoke on social media.

Accounts based in Washington Tweeted a higher percentage of annual total Tweets (44% vs. 39% for Oregon-based accounts) between January-August, suggesting these accounts may have taken a more proactive communication approach prior to the two-month peak period in wildfire smoke compared to Oregon-based accounts. Proactive communication is viewed as critical for helping households prepare for wildfire smoke and effectively mitigate health harms associated with smoke exposure [29]. Washington-based accounts also appeared to prioritize wildfire and smoke messaging that encouraged the adoption of protective actions and informed people about risks using verbal, numeric and AQI formats. On the other hand, accounts in Oregon seemed to prioritize messaging that focused more on community-building by using more community language per Tweet. These differences in communication strategies could be intentionally tailored to their respective populations, or they may simply reflect differences in resources (e.g., trained communications staff) allocated to communications and smoke information dissemination. Future research could offer significant insights into these state-by-state differences by conducting interviews with communications staff and personnel to gather more information.

### Practical implications for public health

The findings of this study can provide numerous lessons to public health practitioners and researchers regarding communication strategies for environmental health hazards like wildfires and smoke. First, our work points to the importance of proactive and sustained communications informing the public about wildfires and smoke *before* they are likely to encounter these hazards. Timely communications are key for educating and empowering individuals to make informed decisions about protecting themselves from harmful exposures. This research also demonstrates how theories of health promotion like the Protection Motivation Theory can be leveraged to craft more effective messages for behavior change. Additionally, we highlight the need for communicators to employ numeric information, verbal cues, and AQI risk labels, to help individuals quantify and compare risk levels across time and space and mitigate impacts on health. Finally, we call attention to the need for communications to increase engagement with communities and for social media to generate more interaction between public health stakeholders and citizens. Although this paper focuses on health messaging related to wildfire and smoke, insights can be drawn for communicating risks associated with exposure to many other environmental hazards.

### Limitations

This research’s main limitations stem from its reliance on Twitter data. In this study, we analyzed Tweets by various governmental institutions in the US that Tweeted in 2022; however, the public likely receives information from numerous other sources (both offline and online), and likely also encounters wildfire and smoke communications on Twitter from accounts that were not captured in our sample. Although similarities likely exist between what an agency Tweets and what they communicate on other channels, our research cannot draw conclusions about all wildfire and smoke communications that the public may view in the US Pacific Northwest; research on other information sources is needed.

It is also important to note that not all individuals use Twitter, and its future as a key governmental communication platform remains uncertain as it has transitioned into a new platform called “X”. Our results may not reflect the current social media landscape and our analysis only captured a limited number of accounts that Tweeted about wildfire and smoke events in the Pacific Northwest region of the US during the study period (2022). Thus, this study examines smoke communication practices on Twitter at a particular snapshot in place and time and future research should be extended to other regions and time periods. We could have used a portion of the total dataset of 85,406 Tweets published between April 2009 and December 31st 2021 and compared these to the January 1st 2022 through December 31st 2022 Tweets that were selected and analyzed for this study to measure changes in messaging from the earlier time period to the time period we selected. However, since earlier time periods did not contain a complete Tweeting history from some accounts, we selected only Tweets from a single year (i.e., 2022) in order to conduct a more in-depth analysis of wildfire smoke communications.

Additionally, we relied on select keywords to score institutions’ use of Protection Motivation Theory dimensions and risk language. Although this approach was informed by prior literature and based on an initial scan of the Tweets, the list of keywords selected likely did not capture an exhaustive list of words that could be associated with the dimensions (or sub-dimensions) scored. We also did not quantify the public’s engagement to Tweets in this research, nor did we measure real behavior change in response to health messaging. Future work could expand on this research considerably by measuring the effectiveness of wildfire smoke communications on behavioral intentions through experimental research. More research is also needed to compare the impacts of communications disseminated during smoke off-seasons versus during peak smoke levels on citizens’ smoke preparedness. Such research on the effectiveness of proactive versus reactive health communications in motivating behavior change would guide health officials in determining the most strategic approach to safeguarding public health during wildfire smoke events. Still, our work offers valuable insights that public health stakeholders can apply to other means of communication that dominate the media landscape going forward.

## Conclusion

Reducing population exposure to wildfire smoke is a major public health challenge in the US Pacific Northwest and effective health messaging is needed to educate the public about smoke-related health risks and how they can mitigate them. This research analyzed Twitter communications about wildfires and smoke from 2022 authored by institutional public health and environmental accounts in Washington and Oregon. This study compared Tweeting patterns over time, in connection with potential wildfire smoke exposure, and evaluated communications based on whether they encouraged the adoption of smoke-protective actions, informed the public about health risks, and promoted community-building. Overall, we found that institutional Tweeting generally coincided with rising wildfire smoke levels, suggesting institutions tailored their messaging in response to potential population exposures to wildfire smoke. This research also found that accounts mostly used Twitter to promote smoke-related behavior change and used it less for the purposes of disseminating wildfire smoke risk information or promoting community-building. This study fills an important knowledge gap around institutional communication practices and social media health messaging about wildfire smoke to highly exposed populations.

### Electronic supplementary material

Below is the link to the electronic supplementary material.

**Supplementary Material 1: Supplemental Table 1.** List of Twitter accounts used to construct Tweet sample for analysis, their characteristics, and the number of Tweets they generated in the year 2022

## Data Availability

The datasets used and/or analyzed during the current study were obtained from Twitter (now ‘X’) using an API, thus, they are not publicly available but are available from the corresponding author on reasonable request.
