# Supplementary material for: Clearing the air: evaluating institutions’ social media health messaging on wildfire and smoke risks in the US Pacific Northwest
Source: BMC Public Health. 2024 Feb 5;24:379. doi: 10.1186/s12889-024-17907-1 (PMC10840270; doi:10.1186/s12889-024-17907-1)
Supplement: Supplementary file 1 — Supplementary Material 1: Supplemental Table 1. List of Twitter accounts used to construct Tweet sample for analysis, their characteristics, and the number of Tweets they generated in the year 2022 [file 12889_2024_17907_MOESM1_ESM.docx]

| **Twitter account** | **Institution type** | **Regional scale** | **State** | **# Tweets (2022)** |
| --- | --- | --- | --- | --- |
| @AIRNow | Environment | Regional | USA | 55 |
| @BFHD | Health | Local | WA | 51 |
| @ChelanDouglasHD | Health | Local | WA | 21 |
| @ClallamHealth | Health | Local | WA | 7 |
| @ClarkCoWA_PH | Health | Local | WA | 17 |
| @EcologyWA | Environment | Regional | WA | 75 |
| @EPAair | Environment | Regional | USA | 31 |
| @EPAnorthwest | Environment | Regional | USA | 49 |
| @GCHD_WA | Health | Local | WA | 2 |
| @HHSRegion10 | Health | Regional | USA | 0 |
| @JoCoPH | Health | Local | OR | 0 |
| @KCPubHealth | Health | Local | WA | 67 |
| @KitsapHealth | Health | Local | WA | 9 |
| @LaneCoPH | Health | Local | OR | 1 |
| @LaneRegionalAir | Environment | Local | OR | 20 |
| @LCPHSS | Health | Local | WA | 11 |
| @MarionCo_Health | Health | Local | OR | 0 |
| @MultCoHealth | Health | Local | OR | 52 |
| [@NWCleanAir](https://twitter.com/NWCleanAir) | Environment | Regional | WA | 549 |
| @OHAOregon | Health | Regional | OR | 28 |
| @OregonDEQ | Environment | Regional | OR | 78 |
| @ORSmokeInfo | Environment | Regional | OR | 88 |
| @pscleanair | Environment | Local | WA | 75 |
| @sjcpublichealth | Health | Local | WA | 1 |
| @SnoHD | Health | Local | WA | 11 |
| @SpokaneCleanAir | Environment | Local | WA | 20 |
| @spokanehealth | Health | Local | WA | 23 |
| @ThurstonHealth | Health | Local | WA | 10 |
| @TPCHD | Health | Local | WA | 14 |
| @WADeptHealth | Health | Regional | WA | 91 |
| @WashcoOregon | Health | Local | OR | 88 |
| @whitman_health | Health | Local | WA | 0 |
| @WWCDCH | Health | Local | WA | 2 |
| @yakimahealth | Health | Local | WA | 8 |

**Supplemental Table 1.** List of Twitter accounts used to construct Tweet sample for analysis, their characteristics, and the number of Tweets they generated in the year 2022.
